# Haploinsufficiency of *Shank3* increases the orientation selectivity of V1 neurons

**DOI:** 10.1038/s41598-022-26402-9

**Published:** 2022-12-23

**Authors:** Carlos Alberto Ortiz-Cruz, Emiliano Jimenez Marquez, Carlos Iván Linares-García, Gerardo Rodrigo Perera-Murcia, Yazmín Ramiro-Cortés

**Affiliations:** División de Neurociencias, Instituto de Fisiología Celular, Universidad Nacional Autónoma de México, Mexico City, Mexico

**Keywords:** Neuroscience, Neural circuits

## Abstract

Autism spectrum disorder (ASD) is a neurodevelopmental disorder whose hallmarks are social deficits, language impairment, repetitive behaviors, and sensory alterations. It has been reported that patients with ASD show differential activity in cortical regions, for instance, increased neuronal activity in visual processing brain areas and atypical visual perception compared with healthy subjects. The causes of these alterations remain unclear, although many studies demonstrate that ASD has a strong genetic correlation. An example is Phelan–McDermid syndrome, caused by a deletion of the Shank3 gene in one allele of chromosome 22. However, the neuronal consequences relating to the haploinsufficiency of Shank3 in the brain remain unknown. Given that sensory abnormalities are often present along with the core symptoms of ASD, our goal was to study the tuning properties of the primary visual cortex to orientation and direction in awake, head-fixed Shank3^+/−^ mice. We recorded neural activity in vivo in response to visual gratings in the primary visual cortex from a mouse model of ASD (Shank3^+/−^ mice) using the genetically encoded calcium indicator GCaMP6f, imaged with a two-photon microscope through a cranial window. We found that Shank3^+/−^ mice showed a higher proportion of neurons responsive to drifting gratings stimuli than wild-type mice. Shank3^+/−^ mice also show increased responses to some specific stimuli. Furthermore, analyzing the distributions of neurons for the tuning width, we found that Shank3^+/−^ mice have narrower tuning widths, which was corroborated by analyzing the orientation selectivity. Regarding this, Shank3^+/−^ mice have a higher proportion of selective neurons, specifically neurons showing increased selectivity to orientation but not direction. Thus, the haploinsufficiency of *Shank3* modified the neuronal response of the primary visual cortex.

## Introduction

Autism spectrum disorder (ASD) is a neurodevelopmental disorder characterized by core symptoms such as impaired communication, social interaction, repetitive or stereotyped behaviors, and sensory alterations^1,7,9,34,62,68^. Furthermore, individuals with ASD exhibit alterations in sensory processing^34,41^. For instance, the performance of children with ASD in identifying a simple shape embedded within a complex figure (Embedded Figure Task; EFT) was at the same level as controls but with reduced activity in cortical areas^37^. In a visual search task, people with ASD showed no differences in performance compared to non-ASD subjects; however, those with ASD showed increased neuronal activity in areas related to visual processing^33^. Similarly, people with ASD showed enhancement activity in areas related to visual perception when they were exposed to objects, faces, and words in a visual perception task^58^. Importantly, quantification of visual attention showed that ASD individuals had a more robust image center bias regardless of object distributions and reduced saliency for faces^71^, besides an EEG study performed in children with ASD (Phelan–McDermid syndrome and idiopathic ASD) displayed abnormal waveforms of visual evoked potentials^64^. All these data strongly suggest that ASD individuals exhibit atypical visual processing. However, the neuronal mechanisms that underlie these alterations remain unknown.

Despite the causes of ASD being myriad, a subset of syndromes are related to genes associated with synaptic structure and function, leading them to be termed “synaptopathies”^6,76^. One of the best characterized examples of this is the Phelan–McDermid syndrome (PMS), caused by a deletion of chromosome 22q13, the outcome which is generally haploinsufficiency of the *Shank3* gene^5,15,20,59^. SHANK3 is a scaffolding protein localized on the post-synaptic excitatory synapses as part of the PSD complex. SHANK3 scaffolds ionotropic and metabotropic receptors by direct or indirect interactions; these receptors are considered key regulators of synaptic transmission and plasticity^39,44,69^. The establishment of proper synaptic function is crucial for normal neuronal activity. Therefore, based on the potential importance of SHANK3 in the synaptic function, it is relevant to understand how the haploinsufficiency of *Shank3* may affect neuronal activity.

Reports from ASD in humans and, recently, in different animal models of neurodevelopmental disorders associated with ASD showed alterations in visual processing^58,64,71^. Recently, evidence of an increased sensitivity of V1 neurons to stimuli with high spatial frequency and low contrast was reported in a mouse model of MeCP2 duplication syndrome^74^. In addition, a reduced percentage of orientation-selective neurons with a broader tuning orientation was reported in a Fragile X mouse model^25^. In both cases, these mutations affected visual cortical processing and behavior.

In this study, we investigate whether the haploinsufficiency of Shank3, an ASD model, alters the neuronal activity of the primary visual cortex (V1) in response to visual stimuli. The Shank3 model is one of the best characterized and established ASD model in different species. To simulate the clinical condition in humans, we used heterozygous Shank3^+/−^ mice. The Shank3^+/−^ mutation deletes the axons 4–9 in the ankyrin repeats domain, which alters the glutamatergic basal synaptic transmission, reduces LTP and GluR1 (an AMPA receptor subunit) expression, reduces social interactions and increases self-grooming^8,73^.

Knowing that the neurons from V1 respond to orientation, direction, contrast and frequency^4,10,21,23,45,51,55,61,66^, we recorded the neuronal activity of V1, specifically L2/3, in response to drifting gratings in wild-type (WT) and Shank3^+/−^ mice. Using in vivo two-photon calcium imaging of GCaMP6f expressed in L2/3 neurons of V1, we analyzed their orientation and direction tuning response to drifting gratings stimuli in head-fixed awake mice. We found a higher proportion of responsive and orientation-selective neurons in Shank3^+/−^ mice in comparison to neurons from wild-type mice. However, no differences were found in the global activity, amplitude, and temporal response profile (ramp index), although neurons from Shank3^+/−^ mice had higher activity and a higher proportion of responsive neurons for specific stimuli. Interestingly, the outcome from orientation tuning showed that neurons from Shank3^+/−^ mice had higher orientation selectivity, while the direction selectivity showed no differences between neurons from Shank3^+/−^ and wild-types. The results outlined above show that the haploinsufficiency of *Shank3* alters the neuronal processing of V1, specifically the orientation selectivity.

## Methods

All experimental protocols were conducted according to current Mexican legislation NOM-062-ZOO-1999 (SAGARPA), and following ARRIVE guidelines^19^, with authorization from the Internal Committee for the Care and Use of Laboratory Animals of the Cell Physiology Institute of UNAM (protocol no. YRC94-16). The experimenters performed data collection and analysis blindly as genotyping was performed post-data processing.

### Animals

B6(Cg)-*Shank3*^*tm1.1Bux*^*/*J heterozygous males (Jax. No. 017889) and C57BL/6J females (Jax. No. 000664) used for breading in our in-house colonies were acquired from The Jackson laboratories. Experiments used B6(Cg)-*Shank3*^*tm1.1Bux*^*/*J males resulting from the backcrossing of B6(Cg)-*Shank3*^*tm1.1Bux*^*/*J male mice into C57BL/6J for at least six generations. Breeding pairs were housed in ventilated cages under a 12 h light/dark cycle, with access ad libitum to food and water. Experimental animals were housed under a cycle of 12 h light/dark, in a conventional temperature and humidity vivarium, with access ad libitum to food and water. Wild-type (Shank3^+/+^, n = 9) and Shank3^+/−^ (n = 9) littermate mice were used for in vivo two-photon calcium imaging.

### Virus injection and cranial window implant

Adult male mice (P60–P75) were anesthetized with 1–2% isoflurane. Under aseptic conditions, a craniotomy (2.5 mm diameter) was made over the left V1 (2.5 mm lateral to the midline, 0.5 mm rostral to lambda) using a dental drill bit ¼” keeping the dura intact. Tissue was maintained hydrous using cortex buffer (NaCl_2_ 125 mM, KCl 5 mM, glucose 10 mM, HEPES 10 mM, CaCl_2_ 2 mM, MgSO_4_ 2 mM, pH 7.4)^31^. Right after craniotomy, viral injections of AAV1-Syn-GCaMP6f-WPRE-SV40 (University of Pennsylvania Vector Core) were performed in V1 (left hemisphere, 2.5 mm lateral to the midline, 0.5 mm rostral to lambda) using a glass micropipette attached to a Nanoject II (Drummond Scientific) at a speed of 4.5 nL per pulse. Injections were made at a depth of 200–250 μm, in three to five different sites (50 nl per site). To prevent the backflow of the virus in each injection during withdrawal, the pipette was kept for over 10 min before retracting it.

After the virus injection, a chronic imaging window was implanted in the craniotomy made of a coverslip (3 mm diameter, #1 thickness) (Warner Instruments, 64-0720). A drop of cortex buffer was applied to fill the gap between the skull and the window, the coverslip was bonded with cyanoacrylate glue (Loctite), and the window was sealed with dental acrylic (Lang dental manufacturing). Finally, a steel head-post^27^ was attached to the skull with the same cyanoacrylate glue and dental acrylic. Eyes were protected and kept moist using ophthalmic ointment (Conforgel, Grin Lab). On the day of surgery, we administered dexamethasone sodium phosphate (i.m. 2 μg g^−1^), lactated ringers solution (s.c. 0.015 ml g^−1^), enrofloxacin (s.c. 5 μg g^−1^) and carprofen (I.p 0.50 mg ml^−1^). Then, enrofloxacin and carprofen were administered for 5 days after surgery. Then, 3–4 weeks after viral injection, animals started the experimental procedures.

### Visual stimulation

Mice became accustomed to the head-fixed station by allowing them to explore the setup for 3 days freely. Next, they were habituated to being head-fixed by fixing them into the station and offering them water and food for 5 days. Each day we increased the time in this mode until they stayed for 30 min with no stress signals, as previously described^27^. Three to four weeks after surgery two-photon calcium imaging was performed. Visual stimuli were generated using custom-written MATLAB (MathWorks) routines using Psychtoolbox. Stimuli consisted of full-field square-wave 4 s drifting gratings (2 cycles/s, 0.0056 spatial frequency, 100% contrast). We used 8 drifting directions separated by 45 degrees presented in sequential order (0, 45, 90, 135, 180, 225, 270, and 315 degrees), recording 5–10 trials for each direction, separated by a 10-s-long gray screen. The stimulation was presented on a 17’’ LCD screen (Dell 17", 60 Hz refresh rate, Dell) positioned 20 cm from the right eye, with a ~ 70° orientation from the mouse nose. Visual stimuli played in Matlab were synchronized with imaging acquisition by custom-written Matlab and Arduino (R3) codes.

### Two-photon calcium imaging

Imaging was performed 3–4 weeks after GCaMP6f injection using a two-photon LSM 710 microscope (Zeiss) based on a galvanometer scanning system controlled by Zen black software. The light source was a Ti:Sapphire laser (Chameleon Ultra II, Coherent) tuned to 900 nm (using 60 to 100 mW at back aperture) through a 20X objective W-Plan Apochromat water immersion (Zeiss, 1.0 NA, 2.4 mm working distance). Images were acquired using the Zen black software at 5 Hz, 512 × 512 pixels, and imaging was performed at a depth of 200–250 μm. Image acquisition and visual stimulation routines were programmed to begin and finish simultaneously using a Zeiss LSM Trigger box and an Arduino UNO board, connected by custom-made circuits and custom-written code in Matlab, Arduino and Python.

### Histology and confocal imaging

**Figure 1:**
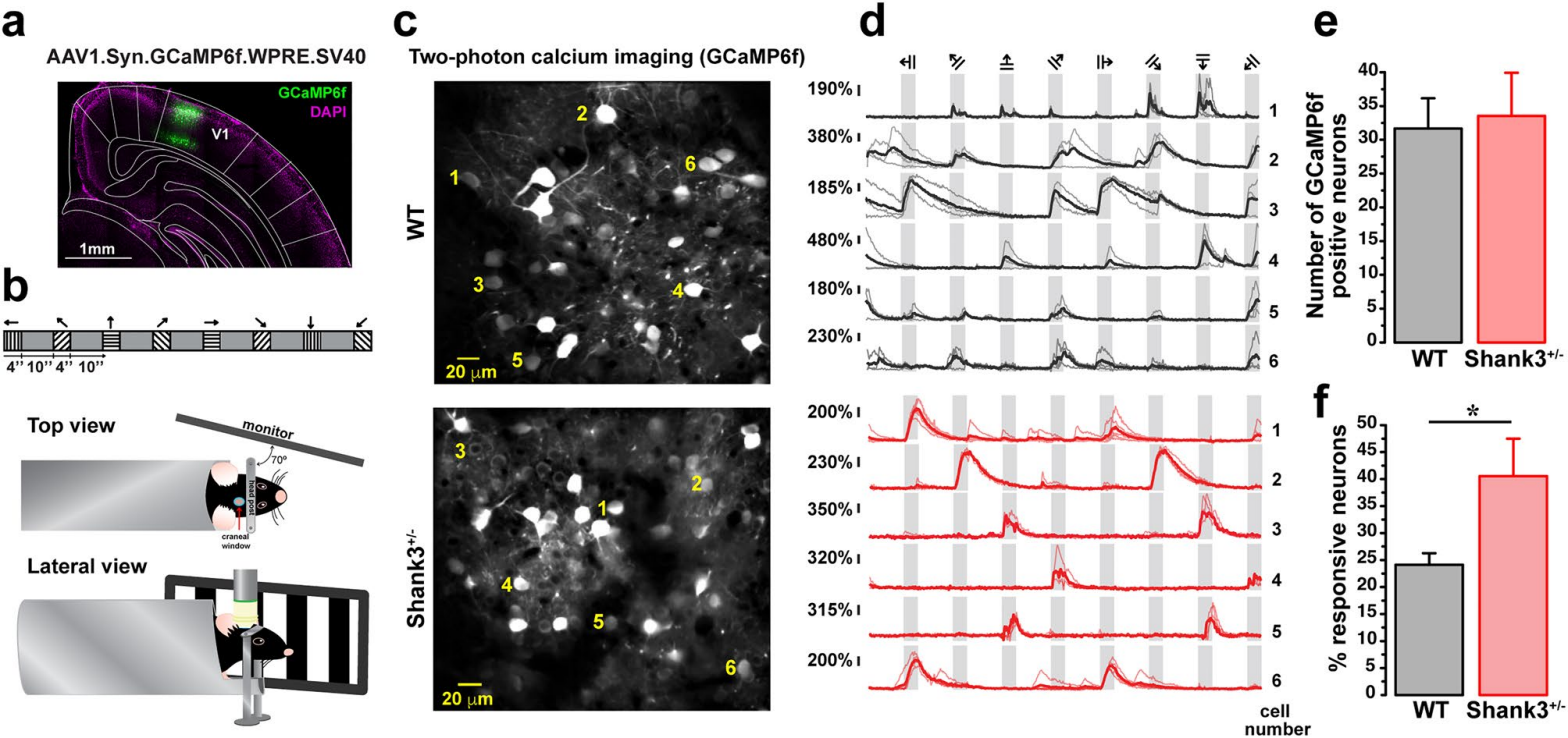
In vivo calcium imaging of L2/3 neurons in V1. (**a**) Representative image of an AAV.Syn.GCaMP6f.WPRE.SV40 injection into V1. (**b**) Cartoon of in vivo two-photon calcium imaging in V1 from head-fixed awake mouse and visual stimuli. (**c**) Representative field of view showing GCaMP6f positive neurons from WT and Shank3^+/−^ mice. Numbers represent examples of somas. Imaging was performed 3–4 weeks after AAV injection, acquired at 5 Hz. (**d**) Calcium transients (∆F/F_0_) of the neurons labeled in (**c**), individual traces (lighter lines), and mean traces (dark lines) are shown from WT (black traces) and Shank3^+/−^ (red traces) mice. Gray background bars indicate the visual stimuli (4 s). (**e**) Quantifying GCaMP6f positive neurons for both genotypes (Student´s *t*-test, *p* = 0.811). Bars represent means ± SEM. (**f**) Percentage of responsive neurons from individual regions of interest in WT and Shank3^+/−^ mice (Student’s *t*-test, * *p* = 0.018). Bars represent means ± SEM.

To verify the injection sites, mice were deeply anesthetized with ketamine/xylazine 85/15 vol/vol, then transcardially perfused with PBS and paraformaldehyde 4% (wt/vol). Brains were fixed overnight in 4% paraformaldehyde and then washed in PBS 1% five times. Coronal slices with 150 μm thickness were obtained using a vibratome S1000 Ted Pella. Confocal images were obtained using an LSM710 (Zeiss) microscope, with a 488 nm laser for GFP excitation, 1024 × 1024 pixels, using an objective 10× C-Apochromat, water immersion, 0.45 NA, 1.8 working distance. We performed tile scans overlapping 10% to construct the reconstruction maps for the infection site (Fig. 1a).

### Image processing and analysis

After image acquisition, the brain motion in raw images was corrected using the cross-correlation image alignment Turboreg plugin (ImageJ). To extract the fluorescence traces (F), we used a constrained non-negative matrix factorization (CNMF) algorithm^50^, choosing somas as the regions of interest (ROIs). ΔF/F_0_ was calculated as (F − F_0_)/F_0_, whereby F_0_ is the baseline fluorescence signal averaged over a 2 s period immediately before starting the visual stimulation. The final calcium transient (∆F/F_0_) to each visual stimulus was the average of five or ten trials. Responsive neurons were considered those with ΔF/F_0_ higher (three standard deviations to basal time, *p* < 0.05) than basal time (2 s before the stimulus) in at least one of the eight stimuli presented. Also, using an AUROC analysis, we classified a responsive neuron when an AUROC between stimulus and baseline fluorescence greater than or equal to 0.8 with 1000 iterations. We determined the preferred orientation (θ_*pref*_) as the stimulus that produced the stronger response. Then we fitted the normalized response tuning curves with a bimodal Gaussian function using the Curve fitting Tool from Matlab:

$$ R\left( \theta \right) = b + c^{{e^{{ - \left( {\frac{{x\left( {\theta - \theta_{pref} } \right)^{2} }}{{2d^{2} }}} \right)}} }} + d^{{e^{{ - \left( {\frac{{x\left( {\theta - \theta_{pref} + 180} \right)^{2} }}{{2a^{2} }}} \right)}} }} $$

where θ_*pref*_ is the preferred orientation, *b* is a constant offset, *c* is the cell's response (ΔF/F_0_) to the preferred orientation, *d* is the response to the orthogonal orientation and, *a* is the tuning width^66^. We measured the goodness of the fit and considered selective neurons, those cells that fit the bimodal Gaussian with an r^2^ > 0.7.

The orientation selectivity index (O.S.I) calculated for selective cells was defined as:

$$ OSI = \frac{{R_{pref} - R_{ortho} }}{{R_{pref} + R_{ortho} }} $$

where the R_*pref*_ and R_*ortho*_ are the response to the preferred and orthogonal orientation respectively.

To characterize the preferred motion direction, we calculated the direction selectivity index (D.S.I) for each cell defined as:

$$ DSI = \frac{{R_{pref} - R_{oppo} }}{{R_{pref} + R_{oppo} }} $$

where R_*pref*_ and R_*oppo*_ are the responses to the preferred motion direction and its opposite, respectively.

To calculate the tuning width for the preferred orientation above the offset, we calculated the full width at half maximum (FWHM) of the bimodal Gaussian function (2√2ln2a)^42,66^. For a better fitting, we constrained the Gaussian parameters as described previously by^42^: *b* was forced to lie in the interval [− M, M], where M is the larger response to any stimulus, *c* and *d* were constrained to lie in the interval [0, 3 M], and width parameter *a* were constrained to lie in the interval [α/16,2α], where α is the step between stimulus (45°). Additionally, we set the star of the fitting using the following initial conditions: *b* = 0, *c* = *e* = *M* and a = α/2.

To quantify the temporal response profile of individual neurons, using only the responsive neurons, we used the ramp index as described by Makino and Komiyama^40^ defined as:

$$ ramp\, index = log_{2} \left( {\frac{R2}{{R1}}} \right) $$

where R1 refers to the mean of ΔF/F_0_ between 1 and 2 s from the visual stimulus onset, and R2 is the mean of ΔF/F_0_ for the last second of the visual stimulus.

### Statistics

Statistics analyses were performed in Matlab using custom-written codes and Origin Pro. Normality analysis (Shapiro–Wilk and Kolmogorov–Smirnov) was performed on all datasets. All tests were performed with a 0.95 confidence level (*p* < 0.05 was considered significant). The area under the receiver operating characteristics (AUROC) analysis was performed to determine if a neuron was responsive to visual stimulation (AUROC > 0.8 between basal fluoresce and fluoresce during the stimuli presentation). The data were bootstrapped (1000 iterations) for AUROC analysis. Student’s *t*-test was performed to determine if the number of responsive neurons differed between groups (Fig. 1e,f).

**Figure 2:**
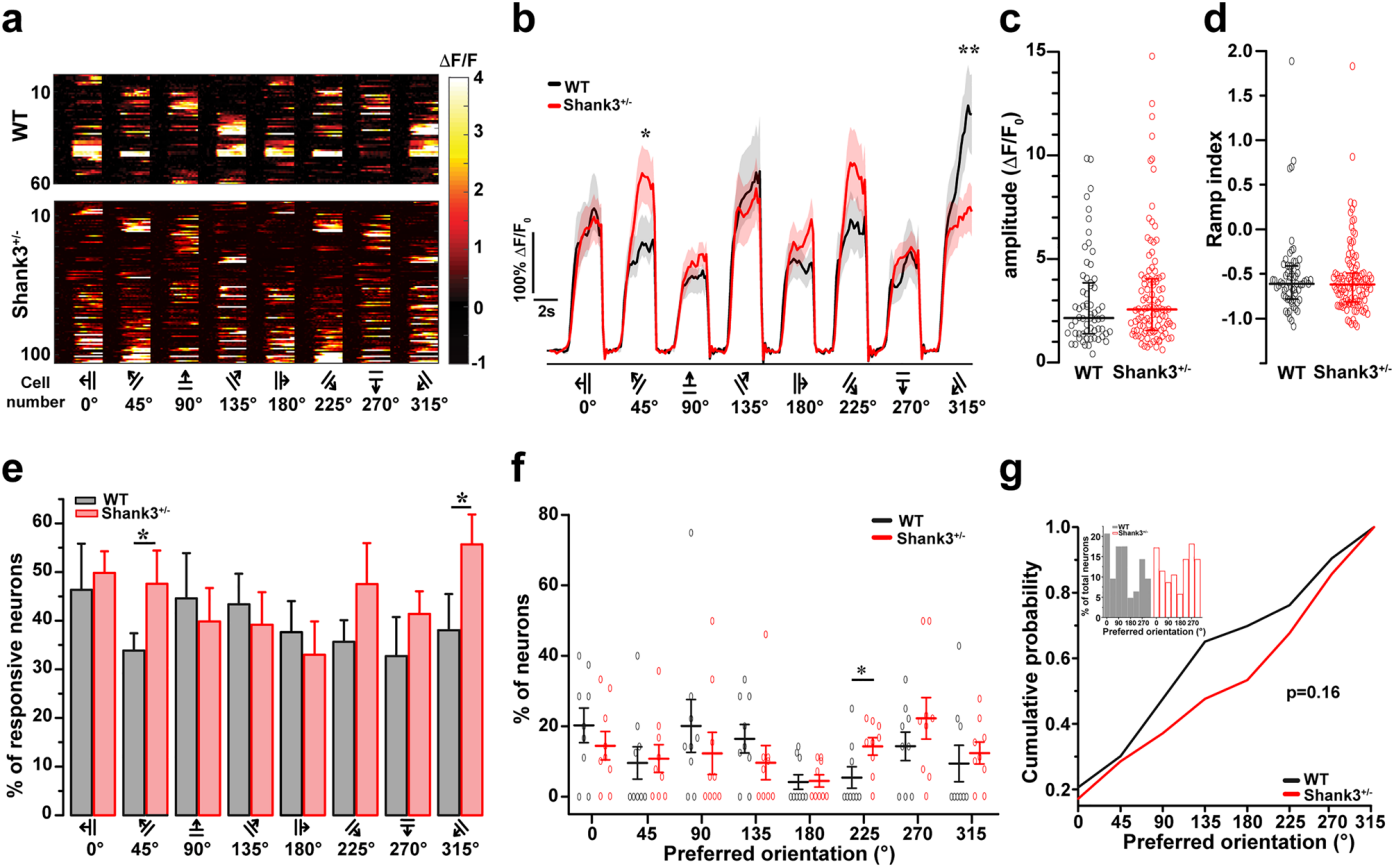
Neurons from Shank3^+/−^ respond differently to specific stimuli. (**a**) Color maps showing each responsive neuron activity evoked by visual stimulation for WT (n = 9 mice, 63 responsive neurons) and Shank3^+/−^ mice (n = 9 mice, 105 responsive neurons). Neurons were sorted by their intensity and their preferred stimulus orientation. (**b**) The mean neuronal activity represented as ∆F/F_0_ evoked for each stimulus in WT and Shank3^+/−^ mice. Lines indicate the ∆F/F_0_ mean values, and lighter shadings show the standard error of the mean. Two-way ANOVA revealed that there was a significant interaction between genotype and stimulus (*p* = 0.0002), followed by Sidak post-hoc test; we found that neurons from Shank3^+/−^ mice showed significant activity differences at 45 degrees (*p = 0.017), and 315 degrees (**p = 0.003). (**c**) Response amplitude from responsive neurons in WT and Shank3^+/−^ mice (Basal to maximum point) (Mann–Whitney U test, *p* = 0.318). Represented as median + IQ range. (**d**) Ramp index from WT and Shank3^+/−^ (Mann–Whitney U test, *p* = 0.594). Represented as median + IQ range. **e**) Proportions of responsive neurons for each stimulus. Student’s t-test, * *p* = 0.046 for 45° and *p = 0.043 for 315°. Bars indicate means ± SEM. (**f**) Percentage of neurons with preferred orientation from WT and Shank3 ± mice. *p = 0.032, Mann–Whitney two-tailed test. Represented as means ± SEM. (**g**) Cumulative probability and histogram for preferred orientation distributions, Kolmogorov–Smirnov, *p* = 0.16.

We used the two-way ANOVA to compare the neuron response intensity for each stimulus (Fig. 2b). To determine if the general intensity of neuronal activity differed between groups, we measured the amplitude of the calcium signal of responsive neurons. We compared them using the Mann–Whitney U test (Fig. 2c). To determine differences in the Ramp index, we used the Mann–Whitney U test (Fig. 2d). We used the Student’s *t*-test to compare the proportion of responsive neurons to each stimulus between groups (Fig. 2e). Mann–Whitney U test was used to determine the proportion of neurons with preferred orientation (measured as maximum ΔF/F_0_) between groups (Fig. 2f). We used Kolmogorov–Smirnov to evaluate whether the distributions of the preferred orientation differed between genotypes (Fig. 2g).

**Figure 3:**
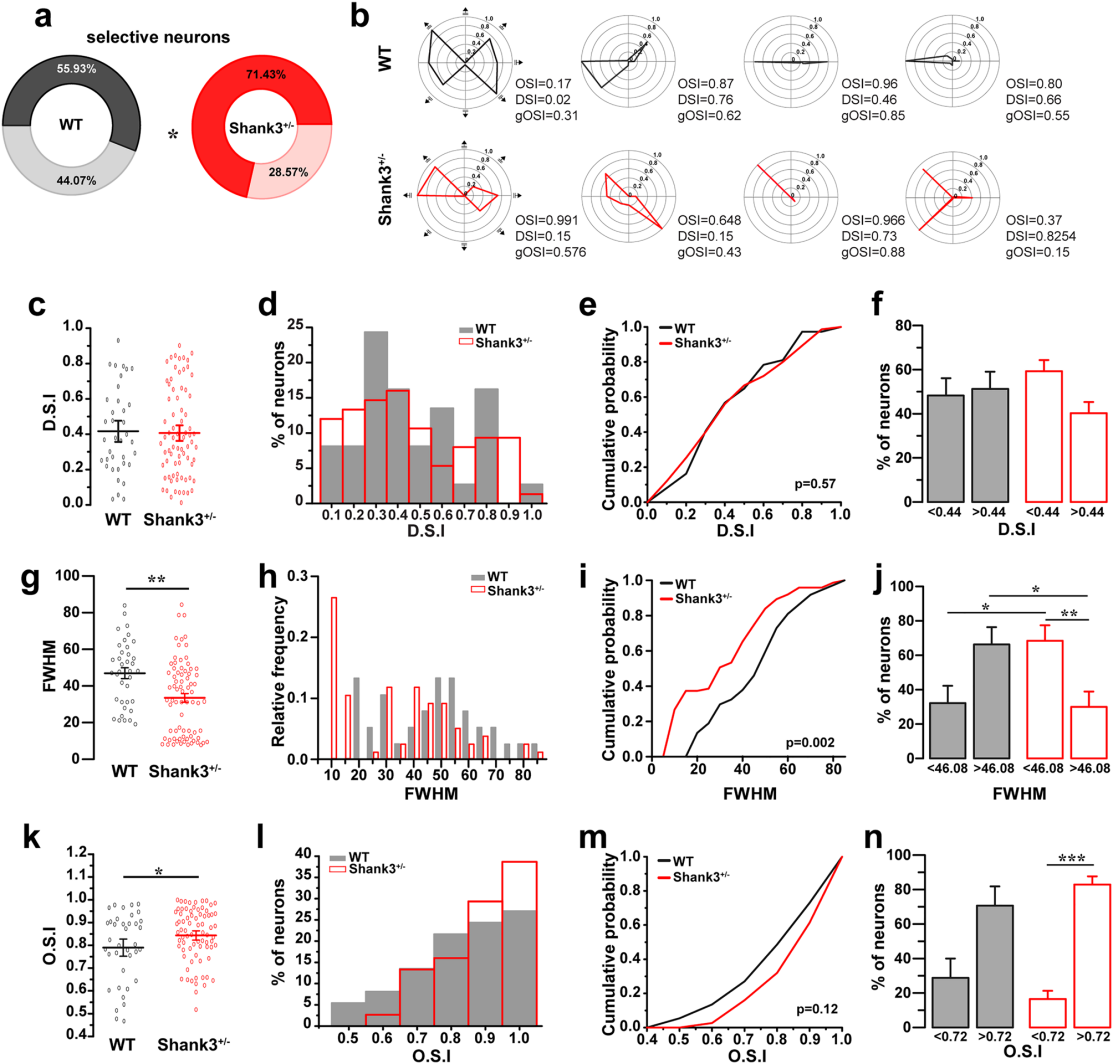
Orientation and direction tuning of V1 neurons from Shank3^+/−^ mice. (**a**) Proportion of selective and non-selective neurons from WT and Shank3^+/−^ mice (Fisher Exact test, * *p* = 0.030). (**b**) Representative polar plots of individual neurons from WT and Shank3^+/−^ mice. Bottom right O.S.I: orientation selectivity index, D.S.I: direction selectivity index, gO.S.I: global orientation selectivity index values. (**c**) Direction selectivity index from V1 in WT and Shank3^+/−^ mice (Mann–Whitney U Test, *p* = 0.881). (**d**) Histogram distributions of D.S.I in V1 neurons from WT (grey bars) and Shank3^+/−^ mice (red open bars). (**e**) Cumulative probability of D.S.I distributions, Kolmogorov Smirnov Test, *p* = 0.57. (**f**) Cutoff of 0.44 for D.S.I. Bars represented as means ± SEM, Kruskal–Wallis test, *p* > 0.05. (**g**) Mean tuning width (FWHM) from selective neurons (Mann–Whitney’s U Test, **p = 0.005). (**h**,**i**) Histogram and Cumulative probability for the distribution of FWHM, Kolmogorov Smirnov Test, *p* = 0.002. (**j**) Cutoff of 0.44 for D.S.I. Bars represented as means ± SEM, Kruskal–Wallis test, *p = 0.03, **p = 0.013. (**k**) Orientation selectivity index from V1 in WT and Shank3^+/−^ mice (Mann–Whitney’s U Test, *p = 0.041). (**l**) Histogram distributions of D.S.I in V1 neurons from WT (grey bars) and Shank3^+/−^ mice (red open bars). (**m**) Cumulative probability of O.S.I distributions, Kolmogorov Smirnov Test, *p* = 0.12. (**n**) Cutoff of 0.72 for O.S.I. Bars represented as means ± SEM, Kruskal–Wallis test, ***p = 0.0018.

We used Fisher's exact test to compare the proportion of selective neurons (Fig. 3a). The D.S.I, FWHM, and O.S.I were evaluated and compared with a Mann–Whitney U test (Fig. 3c,g,k). To calculate the cumulative probability from a histogram of D.S.I, FWHM and O.S.I, we used the Kolmogorov–Smirnov test (Fig. 3e,i,m). A Kruskal–Wallis test was used to analyze the cutoff comparison from D.S.I, FWHM and O.S.I (Fig. 3f,j,n).

### Ethics approval and consent to participate

All experimental protocols were conducted according to current Mexican legislation NOM-062-ZOO-1999 (SAGARPA) and following ARRIVE guidelines^19^, with authorization from the Internal Committee for the Care and Use of Laboratory Animals of the Cell Physiology Institute of UNAM (Protocol No. YRC94-16). The experimenters performed data collection and analysis blindly as genotyping was performed post-data processing.

## Results

To determine whether *Shank3* haploinsufficiency affects the tuning properties of V1, we performed in vivo two-photon calcium imaging in L2/3 of the primary visual cortex (V1) in awake head-fixed mice, comparing wild-type (Shank3^+/+^) with Shank3^+/−^. Neuronal activity in V1 was elicited by visual stimulation (drifting gratings at 100% contrast).

### Responsive neurons are increased in Shank3^+/−^ mice

To monitor the neuronal activity of V1 neurons, we expressed GCaMP6f in L2/3 of V1 through an adeno-associated virus (AAV1-Syn-GCaMP6f-WPRE-SV40)^13^ and performed a cranial window (Fig. 1a). Three to four weeks later, mice were habituated to head fixation to minimize movement during imaging sessions, and the residual motion was corrected (see methods). Then, we recorded the neuronal activity by measuring the somatic calcium responses to sensory stimulation consisting of drifting gratings presented to the contralateral eye in eight angles and directions (Fig. 1b).

Two-photon imaging revealed visual stimulus-evoked calcium transients measured by somatic fluorescence changes (Fig. 1c,d). To confirm that the expression of GCaMP6f was not influenced by genotype, we quantified the number of GCaMP6f positive neurons. No differences were found between WT (31.66 ± 4.47 cells per mouse, n = 9 mice) and Shank3^+/−^ (33.55 ± 6.37 cells per mouse, n = 9 mice, *p* = 0.811) (Fig. 1e). Nevertheless, analyzing the GCaMP6f signals we found an increased number of responsive neurons in Shank3^+/−^ mice (40.55 ± 6.45%, 63 neurons) compared to WT mice (24.12 ± 2.15%, 105 neurons, *p* = 0.018,) (Fig. 1f). These data show that Shank3^+/−^ mice exhibit more responsive neurons and that this is independent of the expression of GCaMP6f.

### Neurons from Shank3^+/−^ respond differently to specific stimuli

Neurons from V1 (L2/3) are endowed with diverse sensitivity to respond to more than one stimulus (Fig. 2a). For instance, pyramidal neurons from V1 respond preferentially to specific stimuli, whereas interneurons have a broader response. We found that neurons from Shank3^+/−^ mice showed more activity than WT for 45 degrees (1.476 ± 0.107 ∆F/F_0_ for WT, 2.169 ± 0.205 ∆F/F_0_ for Shank3, *p* = 0.017), whereas WT neurons responded more strongly than Shank3^+/−^ to 315 degrees (2.666 ± 0.279 ∆F/F_0_ for WT, 1.872 ± 0.158 ∆F/F_0_ for Shank3, *p* = 0.003) (Fig. 2b). No differences were found in the amplitudes from calcium transients to all stimuli (2.155 + IQ for WT and 2.574 + IQ for Shank3^+/−^, *p* = 0.318) (Fig. 2c). Regarding the response temporality, we found a negative ramp index for WT and Shank3^+/−^ without differences between genotypes (− 0.607 + IQ for WT, − 0.615 + IQ for Shank3^+/−^, *p* = 0.594) (Fig. 2d). We examined the proportion of responsive neurons for each stimulus; regarding this, we found a higher percentage of responsive neurons at 45° (33.86 ± 3.55% for WT, 47.60 ± 6.82% for Shank3^+/−^, *p* = 0.046) and 315° for Shank3^+/−^ mice (38.03 ± 5.56% for WT, 61.76 ± 4.39% for Shank3^+/−^, *p* = 0.043); however, the percentage of neurons was unchanged for the other stimuli (Fig. 2e). Analyzing the preferred orientation, most stimuli found no differences between genotypes. However, Shank3^+/−^ mice show a higher percentage of neurons that respond preferentially to 225 degrees (14.19 ± 2.48%) in comparison to WT mice (5.47 ± 3.00%, *p* = 0.032) (Fig. 2f). In line with this, we found no differences between genotypes for the distributions of the percentage of neurons with preferred orientation analyzed by cumulative probability, *p* = 0.16 (Fig. 2g). These results indicate that Shank3^+/−^ mice respond differently to some specific stimuli.

### Orientation tuning is enhanced in Shank3^+/−^ mice

To further examine how the haploinsufficiency of *Shank3* might alter the tuning of V1 neurons, we characterized the tuning properties of L2/3 neurons for orientation and direction. Compared with WT (55.93% of selective neurons), Shank3^+/−^ mice presented a higher proportion of selective neurons (71.43% of selective neurons, *p* = 0.030) (Fig. 3a). However, we identified neurons with broader and narrower responses in both genotypes (Fig. 3b). No differences were found in the direction selectivity (D.S.I, direction selectivity index), where for WT, the mean was 0.416 ± 0.04 D.S.I and for Shank3^+/−^, the mean was 0.406 ± 0.02 D.S.I (*p* = 0.881) (Fig. 3c). Additionally, no differences were found in the proportion of neurons for D.S.I (median D.S.I = 0.367 for WT, median D.S.I = 0.355 for Shank3^+/−^) (Fig. 3d). In line with this, we found that cumulative probability was not different between genotypes (*p* = 0.57) (Fig. 3e). Using a cutoff of 0.44, no differences were found between genotypes for D.S.I < 0.44 (48.52 ± 7.64% for WT, 59.52 ± 4.87% for Shank3^+/−^, *p* > 0.05) and D.S.I > 0.44 (51.48 ± 7.64% for WT, 40.48 ± 4.87% for Shank3^+/−^, *p* > 0.05) (Fig. 3f).

On the other hand, analyzing the tuning-curve sharpness using full-width at half-maximum (FWHM), neurons from Shank3^+/−^ mice show narrower tuning widths (33.51 ± 2.36 mean of FWHM, *p* = 0.005) than WT mice (46.99 ± 2.93 mean of FWHM) (Fig. 3g). In line with this, analyzing the distribution of FWHM (48.09 median for WT, 32.30 median for Shank3^+/−^) (Fig. 3h), we found that cumulative probability was shifted to narrower tuning widths for Shank3^+/−^ mice in comparison to WT mice (*p* = 0.002) (Fig. 3i). In addition, analyzing a cutoff of 46.08, neurons from Shank3^+/−^ mice have a higher proportion of neurons with narrower tuning widths < 46.08 (69.23 ± 8.20%, *p* = 0.030) in comparison to WT mice (32.96 ± 9.35%), by contrast, neurons from WT mice shown higher proportions of neurons with broader tuning widths (67.04 ± 9.35%) in comparison to Shank3^+/−^ mice (30.77 ± 8.20%, *p* = 0.032) (Fig. 3j).

Finally, we analyzed the orientation-selectivity. Neurons from Shank3^+/−^ mice exhibited higher O.S.I (mean 0.843 ± 0.01) compared with WT mice (mean 0.789 ± 0.02, *p* = 0.041) (Fig. 3k). No differences were seen in the proportions of neurons for the orientation selectivity index (O.S.I) between genotypes (median OSI = 0.80 for WT) and (median OSI = 0.85 for Shank3^+/−^) (Fig. 3l). In addition, the cumulative probability was not significantly different (*p* = 0.12) (Fig. 3m). Using a cutoff of 0.72, no differences were found between genotypes for O.S.I > 0.72 (70.93 ± 10.99% for WT and 83.19 ± 4.48% for Shank3^+/−^, *p* > 0.05) and O.S.I < 072 (29.07 ± 10.99% for WT and 16.81 ± 4.48% for Shank3^+/−^, *p* > 0.05). We found a higher proportion of neurons with O.S.I > 0.72 in comparison to O.S.I < 0.72 in Shank3^+/−^ mice (*p* = 0.0018) (Fig. 3n). These data demonstrate that neurons from Shank3^+/−^ mice have higher orientation selectivity than wild-type mice, but the proportion of neurons with high O.S.I is not different from WT mice.

## Discussion

Atypical sensory experience is a ubiquitous feature of autism spectrum disorders (ASD)^56,60,67,68^. It is estimated to occur in more than 90% of autistic individuals. For instance, it has been reported that autistic individuals display atypical visual attention and enhanced visual functioning^24^^,^^58^^,^^71^. Recent works in animal models of neurodevelopmental disorders associated with ASD, such as Fragile X syndrome (a model for mental retardation), have indicated orientation-tuning deficits in V1 neurons^25^. In MECP2 duplication syndrome, also associated with ASD, higher visual acuity and contrast sensitivity in neurons from V1 was described^74^. It is worth mentioning that these two models are considered two monogenic neurodevelopmental disorders, whereby ASD may not be considered a core symptom but may have a high prevalence^3,52,53,70^. These reports support the idea that there might be alterations in visual processing in neurodevelopmental disorders. However, it remains unknown how the visual cortex process visual stimuli and whether tuning properties change in ASD. Herein we used heterozygous Shank3 (Shank3 ±) mice as a model of ASD, taking advantage of the fact that haploinsufficiency of Shank3 in humans causes the Phelan–McDermid syndrome, considered a syndromic form of ASD^17,48,^^59,65,72^. Moreover, it has been demonstrated that haploinsufficiency of Shank3 in mice promotes an autistic-like phenotype with reduced social interaction, increased stereotyped behaviors, altered ultrasonic vocalizations, and synaptic responses^8,73^. Using two-photon imaging in vivo, we characterized orientation and direction tuning in V1 neurons from Shank3^+/−^ mice. Our results show for the first time that the haploinsufficiency of Shank3 increases the orientation tuning response while the direction response remains unaffected.

We found that Shank3^+/−^ mice have more responsive neurons to gratings in layers 2/3 of V1. Furthermore, we observed that neurons of the Shank3^+/−^ show a higher magnitude of GCaMP6f signals to specific angles. Importantly, although only two specific stimuli showed a statistical difference, several incentives showed minor differences in the proportion of cells responding to specific stimuli. These differences may be due to an imbalance of excitation/inhibition in V1. For instance, it has been reported in Shank3B^−/−^ mice that a reduction of GABAergic activity promotes hyper-reactivity and a higher proportion of excitatory responsive neurons in the somatosensory cortex^12^. This is in line with a previous report in the same model (Shank3B^−/−^ mice), where the expression of PV was reduced in the prefrontal cortex^22^. Besides, evidence demonstrates a reduction in glutamatergic transmission or expression of glutamatergic receptors in different brain structures from Shank3^−/−^ mice^22,29^ or humans PSC^14,63^. In addition, a consequence of this disruption of glutamatergic transmission may be due to alterations in the morphology of dendrites or dendritic spines, which has been reported in Shank3 knockout mice and Shank3 deficient humans neurons^14,26,32,47^ which can modify the synaptic response^49^. Altogether these data suggest a disruption in the excitation/inhibition balance and structural correlates in the Shank3 model. Nevertheless, it is worth noting that we used heterozygous mice instead of knockouts, and still, the level of GABAergic and glutamatergic activity in the Shank3^+/−^ mice model remains unknown.

To characterize the tuning properties of V1 in Shank3^+/−^ mice, we analyzed the direction and the orientation selectivity. The direction tuning of V1 cells in Shank3 ± mice remained unaltered, suggesting that ganglion cells on the retina of Shank3 ± mice may not be altered since it is known that direction selectivity in mice is encoded by these cells and is independent of experience^16,18,54,55^. Furthermore, the retinogeniculo-cortical pathway that refines the direction selectivity during development must also be unaltered in Shank3^+/−^ mice^11,30,55^. Our findings demonstrate that despite the haploinsufficiency of *Shank3*, the intrinsic process that computes direction in V1 is not altered.

In contrast, analyzing the orientation tuning, we found narrower tuning widths and a higher orientation selectivity index in Shank3^+/−^ mice compared to WT. The orientation selectivity comes from dLGN providing tuned inputs to V1, where a substantial proportion of orientation-selective retinal ganglion cells have been reported^66,75^. Additionally, data suggest that orientation selectivity is inherent to dLGN^61^, but could also depend on the thalamocortical circuit, which sends tuned inputs to L4 and this layer sends inputs to L2/3^35,45^, that common dLGN axons preferentially innervated L4→L2/3 connected pairs^43^. Taking this information into account and considering that the direction selectivity was not altered in Shank3^+/−^ mice, our data suggest that the computation of orientation selectivity may be affected by dLGN→L4→L2/3 neuronal subcircuits in the Shank3^+/−^ mice. However, the mechanism that underlies the increased orientation selectivity in Shank3^+/−^ mice remains to be elucidated. One possibility may be the activity of PV cells in Shank3^+/−^ mice since it has been reported that PV activation in awake mice significantly improves the orientation tuning of V1^38^. Another attractive explanation for the increased orientation selectivity in Shank3^+/−^ mice may be asynchrony in inputs that converge onto a cortical neuron, like a random connectivity model^46^. It would be interesting to study the activity of PV neurons in Shank3^+/−^ mice because, as we mentioned before, Shank3B knockout mice have reduced activity in PV interneurons leading to hyper-reactivity in the somatosensory cortex^12^, which might trigger the asynchrony of inputs on V1. Nevertheless, there is controversy about the participation of PV cells in the tuning properties of V1 pyramidal cells that must be considered since it has been reported that the inhibition of PV neurons has no impact on the tuning properties of V1^2^. Furthermore, it becomes essential to consider the balance excitation/inhibition in the orientation selectivity due to the selectivity becoming strong when this balance occurs. Also, it is known that excitatory and inhibitory presynaptic ensembles are co-tuned for the orientation^28,36,57^. Altogether, here we show that the haploinsufficiency of *Shank3* alters orientation selectivity but does not affect direction selectivity, strongly suggesting that the alteration may be in the cortical processing independent of the retinal processing.

## Conclusion

In summary, we demonstrate that the haploinsufficiency of *Shank3* alters the neuronal activity of neurons in L2/3 from V1. We show that Shank3^+/−^ mice have a bigger proportion of responsive neurons to drifting gratings, and these neurons respond differently to specific stimuli. Analyzing the tuning properties in response to drifting gratings, where the stimulus presented changes in orientation and direction, we found that neurons from Shank3^+/−^ have narrower tuning widths and higher orientation selectivity. Interestingly, we did not find differences between Shank3^+/−^ mice and WT mice regarding direction selectivity. Thus, our data suggest that the cortical processing is altered due to *Shank3* haploinsufficiency without affecting the retinal processes that encode the direction selectivity in mice.

## Data Availability

All data generated or analyzed during this study are included in this published article.

## Abbreviations

ASD: Autism spectrum disorder
PMS: Phelan–McDermid syndrome
V1: Primary visual cortex
LTP: Long-term potentiation
AMPA: α-Amino-3-hydroxy-5-methyl-4-isoxazole propionic acid receptor
GCaMP: Green fluorescent protein calmodulin peptide
PSD: Postsynaptic density
L2/3: Layer 2 and 3
EFT: Embedded figure task
EEG: Electroencephalogram
CNMF: Constrained nonnegative matrix factorization
ROI: Regions of interest
AUROC: Receiver operating characteristic
O.S.I: Orientation selectivity index
D.S.I: Direction selectivity index
FWHM: Full width at half maximum
WT: Wild-type
dLGN: Dorsolateral geniculate nucleus
PV neurons: Parvalbumin-positive neurons
AAV: Adeno-associated virus
